# New Molecular Players in the Development of Callosal Projections

**DOI:** 10.3390/cells10010029

**Published:** 2020-12-26

**Authors:** Ray Yueh Ku, Masaaki Torii

**Affiliations:** 1 Center for Neuroscience Research, Children’s Research Institute, Children’s National Hospital, Washington, DC 20010, USA; 2 Department of Pediatrics, Pharmacology and Physiology, School of Medicine and Health Sciences, The George Washington University, Washington, DC 20052, USA

**Keywords:** corpus callosum, callosal projections, cerebral cortex, development, cortical neurons

## Abstract

Cortical development in humans is a long and ongoing process that continuously modifies the neural circuitry into adolescence. This is well represented by the dynamic maturation of the corpus callosum, the largest white matter tract in the brain. Callosal projection neurons whose long-range axons form the main component of the corpus callosum are evolved relatively recently with a substantial, disproportionate increase in numbers in humans. Though the anatomy of the corpus callosum and cellular processes in its development have been intensively studied by experts in a variety of fields over several decades, the whole picture of its development, in particular, the molecular controls over the development of callosal projections, still has many missing pieces. This review highlights the most recent progress on the understanding of corpus callosum formation with a special emphasis on the novel molecular players in the development of axonal projections in the corpus callosum.

## 1. Introduction—The Corpus Callosum

**Figure 1:**
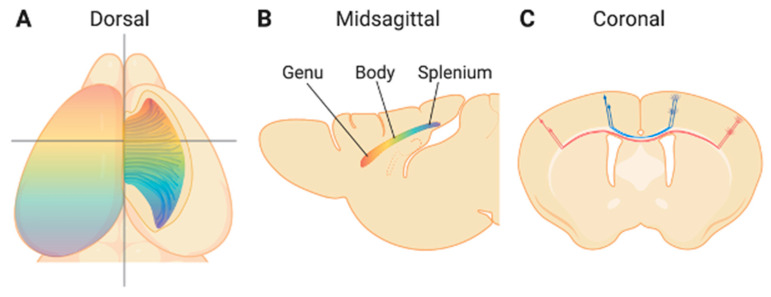
The organization of the mouse corpus callosum (CC). Dorsal (**A**), midsagittal (**B**), and coronal (**C**) views of the CC. Callosal axon projections from each cortical hemisphere cross the midline and primarily connect with homotopic cortical regions in a topographic manner, by which projections from anterior and posterior cortical regions form anterior and posterior parts of the CC, respectively (represented by rainbow colors in **A** and **B**). Callosal projections from the medial and lateral regions of the cortex form the dorsal and ventral portions of the CC, respectively (**C**).

The corpus callosum (CC) is the largest white matter tract that connects two cerebral hemispheres of placental animals. The communication via approximately two hundred million callosal axons allows efficient information exchange between the two hemispheres, coordinating our higher-order motor, sensory, and cognitive tasks [1,2]. The human CC is anatomically divided into several regions that topographically connect two hemispheres: The anterior-most rostrum, genu, body, isthmus, and splenium at the posterior end, while the mouse CC is usually divided into three regions: genu, body, and splenium (Figure 1A,B). Along the anterior–posterior axis, the genu and rostrum connect the frontal and premotor regions of the cerebral cortex, the body conjoins the motor, somatosensory, and parietal regions, while the splenium links the temporal and occipital cortices on both sides [3,4,5]. Although the organization of the CC is defined anatomically, corresponding functional topography has been found based on imaging studies and studies on patients who underwent callosal resection, as well as studies using animal models. For example, neuronal signals for the motor function pass through the genu, while somatosensory inputs go through posterior body of CC. Axons in isthmus are in charge of transmitting auditory signals, and visual information via splenium [6,7]. Dorsal and ventral parts of the CC then connect the medial and lateral cortical regions, respectively [8,9,10] (Figure 1C). Different parts of the CC, therefore, consist of axonal projections from different cortical regions. In addition, callosal projections from each cortical region also include axons of cortical neurons in multiple cortical layers [11,12,13]. It is estimated that 80% of callosal axons come from neurons in layer II/III, and 20% are from neurons in layer V in the mature brain [11].

With these highly precise neuronal connections, the CC integrates inputs from left and right sides of the body for central processing and coordinates bimanual motor movements [14]. The presence of an intact, functional CC facilitates signal exchange between the two hemispheres [15]. The integrity and the efficiency of interhemispheric information exchange is also strongly correlated with social-cognitive functions [16,17,18]. As such, if the formation of CC is compromised (e.g., agenesis, dysgenesis, or hypoplasia) by genetic mutations or prenatal exposure to environmental insults [19,20,21,22,23], the abnormal CC leads to various transient and permanent symptoms. These include impairment in problem solving, dyslexia, ataxia, apraxia, alien limb, agraphia, paresis, and mutism [1,24]. Understanding the mechanisms of CC development is therefore critical to maintain the health and wellbeing of human life.

In the following sections, we will first review the findings on prenatal development of callosal projection, and move onto the less understood postnatal processes of callosal projection development. As this review focuses on the molecular mechanisms underlying these events, we leave out some important topics on CC development, including the formation of midline glial structure, myelination, and activity-dependent refinement, and instead only present informative reviews on these topics.

## 2. Prenatal Development of Callosal Projections

The formation of the CC begins with the projection of pioneer fibers from the cingulate and rostrolateral cortices [25,26,27] at around the 12th week of gestation (GW) in humans, followed by formation of the genu [20,28]. The fusion of these different parts of the CC completes at around GW14, and the CC expands as the brain increases in size [29,30]. Though each region grows at different rates, the overall volume of the CC increases rapidly in early development (GW19–21), and continues to grow at a slower rate until it reaches a plateau at around GW33 [29,31,32].

Our current understanding of prenatal development of the CC at the molecular and cellular levels have mainly come from studies using animal models such as mice, cats, and monkeys, while studies of fetal specimens have supplied the anatomical understanding of CC development in humans. Since there are many excellent reviews on the prenatal development of the CC available already (e.g., [11,33,34]), we will review this topic focusing on the latest discoveries.

### 2.1. Specification of Callosal Projection Neurons

Callosal projections in the mature brain consist of axons of pyramidal neurons in layer II/III and layer V, and to a lesser extent, of layer VI [21,35]. These cortical neurons that project axons to the contralateral hemisphere through the CC are callosal projection neurons (CPNs). CPNs project within the telencephalon, and are further classified based on their connecting target(s): (1) CPNs that only have one projection to the contralateral cortex, (2) CPNs that project to contralateral cortex and one of the striata, and (3) CPNs that have dual projections to contralateral cortex and ipsilateral frontal cortex [35]. Consistent with the general order of cortical layer development, CPNs in deeper layers (layer V/VI) are born and project to the contralateral target earlier than those in superficial layers (layer II/III) [11,26,36,37].

#### 2.1.1. SATB2-mediated Specification

**Figure 2:**
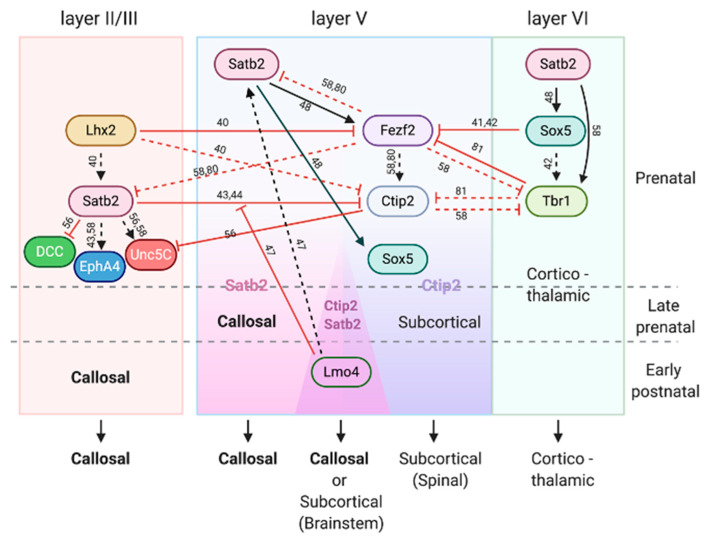
Interactions between key transcription factors and axon guidance molecules for CPN specification. SATB2-associated interactions during prenatal period define TBR1^+^ corticothalamic projection neurons in layer VI, SATB2^+^ CPNs in layer II/III, as well as SATB2^+^ CPNs and CTIP2^+^ subcortical (spinal) projection neurons in layer V. LMO4 further specifies CPNs or subcortical (brainstem) projection neurons from SATB2^+^/CTIP2^+^ populations during the early postnatal period in layer V. Regular arrows and flat-headed arrows indicate positive and negative regulations. Continuous lines indicate direct regulations, while dashed lines indicate suggested interactions demonstrated by knockout mouse and/or in utero electroporation experiments. Reference numbers supporting each interaction are indicated.

Specification of CPNs has been a topic of strong interest. SATB2, a protein involved in transcription regulation, is probably the most-studied molecule for CPN development. Many findings on the molecular mechanisms for specification of CPNs have been obtained through studies on the role and function of SATB2 [38,39]. Figure 2 summarizes the interactions between key transcription factors and axon guidance molecules (will be discussed in the next section) for the fate determination of CPNs and other projection neurons during the prenatal as well as early postnatal (will be discussed later) period [40,41,42].

SATB2 plays a central role in defining the identity of CPNs in the developing brain. In seminal works by Britanova et al. and Alcamo et al., they independently utilized different *Satb2* knockout (KO) mouse models to investigate the role of SATB2 on CPN specification and callosal projections [43,44]. Both groups reported that *Satb2* KO mice failed to form the CC, and the loss of *Satb2* in upper layer neurons led to expanded expression of CTIP2 (also known as BCL11B), a transcription factor that is expressed in subcortical projection neurons in deep layers and plays an essential role in their specification [43,44]. Such a transcriptional network is well conserved across mammalian species [45]. A protooncogene *Ski* cooperates with SATB2 in the direct transcriptional repression of *Ctip2* [46]. Accordingly, *Ski* KO mice result in complete agenesis of the CC akin to *Satb2* KO mice due to rerouted projection to the corticospinal tract or decreased CPN population [46].

Importantly, it has recently been shown that SATB2 is not necessary only for callosal projections, but also for sub-cerebral projections [47,48]. SATB2 promotes sub-cerebral projection neuron identity in layer V by directly activating transcription of *Fezf2* and *Sox5*, while suppressing sub-cerebral projection neuron characters in CPNs in upper layers [48]. FEZF2 in turn negatively regulates SATB2 to suppress CPN characters in layer V sub-cerebral projection neurons [48]. Thus, SATB2 plays its roles in promoting these two different neuronal populations in a cell context-dependent manner.

In addition to these transcriptional regulations, expressions of SATB2 and CTIP2 are partially controlled by calcium signaling, histone methylation, and other mechanisms. Studies on Timothy Syndrome (TS), which is caused by a mutation in the L-type calcium channel Ca_v_1.2, shed light on the importance of calcium on CPN differentiation. The TS mutation prohibits necessary expressional change of two Ca_v_1.2 isoforms during early postnatal corticogenesis. Paşca et al. first pointed out the altered ratio of SATB2 and CTIP2 in neuronal cultures from TS patient-derived induced pluripotent stem (iPS) cells [49]. This observation has been followed up recently using in utero electroporation in mice [50]. The expression of mutated Ca_v_1.2 in developing mouse brains reduced the expression of SATB2 and increased the expression of CTIP2. The altered ratio of SATB2 and CTIP2 expressions, therefore, may be due to the sustained calcium elevation resulting from the mutation.

Methyl transferase DOT1L has been previously reported to affect callosal development via interacting with AF9, a protein involved in transcription regulation [51]. It was recently shown that DOT1L-deficiency led to downregulation of SATB2 and upregulation of CTIP2 in embryonic mouse brains [52]. Chromatin remodeling mediated by an ATP-dependent chromatin remodeling factor SNF2H is required for embryonic expansion of intermediate progenitor cells and for the following specification of SATB2^+^ CPNs in the developing cerebral cortex. Telencephalon-specific knockout of *Snf2h* causes partial agenesis of the CC due to reduction of SATB2^+^ upper layer CNPs [53]. Inositol polyphosphate 4-phosphatase II (INPP4B), a PI (3, 4) P2 metabolizing 4-phosphatase, was another molecule found to regulate the formation of SATB2^+^ pyramidal neuron population and control callosal axon polarization, although the detailed mechanism has yet to be explored [54].

Timing of SATB2 expression may also be a critical factor for CPN fate specification. Premature SATB2 overexpression in the mouse cerebral cortex steers CPNs to acquire a marsupial-like projection fate, instead sending axons through the anterior commissure [45], which is phylogenetically the oldest of forebrain commissures. A recent study has similarly provided evidence that the peak birthdate of CTIP2^+^ laterally-projecting neurons in layer V is slightly earlier than that of SATB2^+^ medially-projection neurons within the same layer V, or even those in layer VI in the embryonic mouse lateral cortex. This observation has suggested a general sequential order in the generation of subcortical (lateral) projection neurons and callosal (medial) projection neurons [55].

The interactions of axon guidance cue Netrin-1 with its attractive and repulsive receptors DCC and UNC5C have been shown to be involved in the formation of callosal projections of deep layer CPNs in mice [56]. Likewise, mutations in *DCC* have been shown to cause agenesis of the CC in humans [57]. *DCC* is directly downregulated by SATB2 [56], while *Unc5C* is directly upregulated and downregulated by SATB2 and CTIP2, respectively [56,58]. SATB2 also directly upregulates the expression of *EphA4*, an Eph family receptor tyrosine kinase that mediates another major axon guidance pathway, Eph/ephrin signaling, in CPNs [58]. In summary, SATB2 and CTIP2 directly contribute to the establishment of specific neuronal connections of callosal and subcortical projection neurons by, at least in part, controlling downstream axon guidance molecules.

#### 2.1.2. Other Players in CPN Specification

Despite decades of hard work described above and in literature, the mechanisms underlying CPN specifications remain to be further elucidated. There has been significant progress in the search for molecules potentially involved in CPN specification [11,35,59]. In pioneering studies by Molyneaux et al. and other groups, comparative microarray analyses on retrogradely labeled and isolated CPNs have identified a number of genes specifically expressed in mouse CPNs at several key embryonic and postnatal stages [36,60,61,62,63,64,65]. Enrichment of many of these genes in lower layer CPNs are highly conserved in the developing primate brain, while genes expressed in upper layer CPNs show greater variability in the degrees of conservation, supporting the hypothesis of evolutionary expansion of upper layer CPN subpopulations in primates [60].

The roles of multiple identified genes in CPN specification are just beginning to be elucidated. One such gene is a transcription factor, *Foxg1* [66,67]. FOXG1 binds to an enhancer site of another transcription factor COUP-TF1, repressing its expression in the mouse somatosensory cortex [68]. Ectopic expression of FOXG1 in layer IV transforms local projection neurons in this layer to acquire pyramidal morphologies, SATB2 expression, and callosal projections, while removal of FOXG1 in layer II/III projection neurons de-represses COUP-TFI and converts them to layer IV neuron identity [68]. FOXG1 has been shown to also form a complex with another transcription factor, RP58, in these layer II/IIII neurons and directly repress the expression of *Robo1* and *Slit3*, components of Slit/Robo signaling in axon guidance [66]. This repression is critical for guiding callosal axons to cross the midline, and inactivation of one allele of *Foxg1* in cortical neurons is sufficient to cause agenesis of the CC [66].

Along with the progress in search of CPN-specifying genes, recent studies have revealed that CPNs in mature brains are more heterogenous than previously thought. RNA in situ hybridization combined with retrograde labeling of cortical neurons in mature mouse cortices has identified various molecules that are expressed in CPN subtypes [69]. More recent high-throughput screenings using bulk and single-cell RNA sequencing further advanced molecular identification of CPN subtypes [64,70]. Highly heterogenous transcriptional identities of both upper layer and lower layer CPNs have been found in these studies. The heterogeneity largely reflects their axonal targets rather than their birth dates or laminar positions [36]. This is in agreement with the finding that target specificity is a strong predictor of molecular identity of long-range projection neurons in layers V and VI [71]. Physiologically, upper layer CPNs have longer action potential duration and lower firing rate, while lower layer CPNs have higher firing rate, but smaller action potential width compared to upper layer CPNs, regardless of their localization [72]. Together, the specification of CPN subtypes during development determines complex combinations of shared and distinct cellular properties between them. Table 1 summarizes the recently reported molecules that are shown to affect CPN specification.

### 2.2. Guidance of Callosal Axons

Guidance of callosal axons is a series of well-orchestrated interactions of attractive and repulsive cues that navigates extending axons to their targets on the contralateral side [21,90,91]. Callosal axons from lower cortical layers cross the midline during the embryonic period, while callosal axons from upper cortical layers do not reach the midline until postnatal stages [92]. The guidance cues for early callosal axon growth are mainly provided by midline glia and pioneering axons [27,93], but other brain tissues, e.g., meninges, or CPNs themselves, also participate in delivering axon guiding cues [94,95].

Guidance of callosal axons mediated by the midline glial structures has been most extensively studied [9,96,97,98,99,100]. The transient glial structure is formed at the midline of the embryonic brain [97,98,99,100,101,102], and consists of the radial glia-derived glial wedges (GW) located by the lateral ventricles [103], the indusium griseum (IG) at the dorsal midline, the midline zipper glia (MZG) at the ventral midline, and the glial slings (GS) that migrate from the ventricles and physically bridge the two hemispheres below the longitudinal cerebral fissure [100]. The GS is actually a migratory population of developing neurons in the subventricular zone [97]. Additionally, subgroups of GABAergic neurons from the ganglionic eminence also migrate into the midline, serving as guidepost cells and attracting projecting axons [97,104]. Since there are other excellent reviews on midline structure development [91,105], we will not discuss it in detail in this review.

Multiple signaling pathways coordinate the complex process of midline axon guidance. In addition to major axon guidance pathways such as Semaphorin/Plexin/Neuropilin, Slit/Robo, Eph/ephrin, and Netrin/DCC/Unc5 pathways, Wnt/Ryk and FGF8/MAPK pathways also partake in the process to a minor degree [106]. Recent studies have been continuously identifying novel components of each signaling pathway and links between them, filling in the missing pieces of the complex jigsaws of signaling required for developing precise interhemispheric connection. Table 2 provides the list of recently reported molecules that are involved in CPN axon guidance.

#### 2.2.1. Players in Semaphorin/Neuropilin/Plexin Pathway

Semaphorins are a family of secreted, transmembrane, or GPI-anchored proteins that are essential for axon guidance and other processes in neural development. Class 3 Semaphorins (SEMA3A and SEMA3C), play important roles in midline guidance of callosal axons through the signaling mediated by their coreceptors formed by Neuropilin-1 and Plexin-A1 [114,115]. In contrast to the classic view, a recent study has shown that SEMA3E signaling mediated by its receptor Plexin-D1 and the adaptor protein GIPC1 plays a role in layer positioning of CPNs, but not in projection of callosal axons [116,117]. Another update on Semaphorin signaling is its crosstalk with ephrin-B1 to control the navigation of post-crossing callosal axons [118]. After midline crossing, callosal axons switch off their response to axon guidance cues including SEMA3C that act as attractants for these axons before crossing the midline. This change is due to the inhibition of SEMA3C signaling by upregulated ephrin-B1 and its interaction with Neuropilin-1 independent of Eph receptors [118].

#### 2.2.2. Players in Slit/Robo Pathway

Slit/Robo signaling plays a crucial role for proper guidance of both pre-crossing and post-crossing callosal axons [102,119,120]. Recently, amyloid precursor protein (APP), which is known to have a central role in Alzheimer’s disease, was identified as a novel receptor of Slit [107]. APP is strongly expressed in the embryonic and neonatal CC and layer V neurons along with other brain regions such as internal capsule, hippocampal commissure, and anterior commissure. Upon binding to Slit, APP transduces intracellular signaling to mediate axon repulsion. Double knockout of *APP* and its family member *APLP2* in mice causes failure of callosal axons to cross the midline [107].

#### 2.2.3. Players in Netrin/DCC/Unc5 Pathway

Netrin-1 can be of attractive or repulsive cue depending on the receptor it binds and downstream intracellular pathways [106,121,122]. Although an extensive amount of knowledge on the mechanisms of Netrin-1-mediated axon guidance has been gained through the research on neuronal projections other than callosal projections, several key findings have been brought from the studies on callosal axon guidance. For example, Netrin-1 has been shown to be attractive for callosal pioneering axons from the cingulate cortex to assist them cross the midline, but not for callosal axons from CPNs. Instead, Netrin-1 prevents SLIT2-mediated repulsion, allowing CPN axons to grow toward and across the midline [123]. Recent studies have added new players in the Netrin-1 pathway; the heat shock cognate protein HSC70 is required for the stability of the DCC/TRIO signaling complex at the growth cone to mediate Netrin-1-induced callosal axon outgrowth and guidance [108]. Similarly, myristoylated alanine-rich C-kinase substrate (MARCKS) mediates Netrin-1-induced DCC activation via membrane recruitment of tyrosine kinases PTK2 and SRC during CC formation [110]. It is also suggested that phospholipase C gamma1 (PLCγ1), a signal transducer of receptor tyrosine kinases, plays a key role in CC formation downstream of SRC kinase by triggering actin rearrangement for axonal growth [109].

## 3. Postnatal Development of Callosal Projections

The postnatal development of the CC is a process as elegant and complicated as the prenatal process. In primates, postnatal CC development completes at a much later stage compared to rodents [124], and this characteristic is considered to involve in the evolution of higher cognitive function [125]. Mass spectrometry-based proteomic profiling of developing mouse CC revealed that many proteins involved in axon growth and guidance reach their peak expression around P3, while proteins for neuronal maturation, glial development, myelin formation, and synapse formation increase their expression after P10, highlighting the dynamic and phased progress of postnatal CC development associated with the expression of a unique set of molecules at each stage [126]. During the early postnatal development of callosal projections, CPNs are further specified and additional callosal projections from upper layer CPNs continue to grow and cross the midline. This section will compile recent findings on the molecular mechanisms associated with these postnatal processes of callosal projection development. Although we do not discuss in this review, important processes of postnatal CC development include morphological and functional maturation of CPNs and their axons, myelination, and activity-dependent neurite and synapse growth and refinement as well [127,128,129,130].

### 3.1. Postnatal Specification of Callosal Projection Neurons

Specification of CPNs that starts from the prenatal period continues postnatally. Various molecules are involved in further establishing their identities, which include specific morphologies and gene expression. Transcription factors have a broad influence on CPN development, since their downstream gene regulation can collectively cover every single aspect of CPN identities. Transplantation of embryonic neurons that have already been fate-restricted to lower layer projection neurons or partially fate-restricted post-mitotic neuroblasts into early postnatal somatosensory cortex results in their integration as projection neuron subtypes with proper molecular and electrophysiological identities. They not only migrate into correct layer positions, but also form appropriate callosal and subcortical axonal projections [131], indicating that the regulations during prenatal period have predominant effects on the establishment of their final specificity.

On the other hand, embryonic and early postnatal CPNs in upper layers can be reprogrammed in vivo into layer V/VI neurons with molecular properties and axonal connectivity of corticofugal projection neurons, by forced expression of FEZF2 [132]. Efficiency of lineage reprograming is high by expressing FEZF2 from embryonic day (E) 17.5, but becomes lower by expressing FEZF2 from postnatal day (P) 3. Moreover, the reprogramming cannot be achieved by expressing FEZF2 after P21 [132]. This suggests that early post-mitotic upper layer CPNs still have plasticity to change their identity, but become progressively more fate-restricted during the postnatal period.

#### 3.1.1. Transcription Factors

Transcription factors that are in effect during prenatal development continue their influence in postnatal stage. SATB2 is one such example. Although SATB2 was initially suggested to be a determinant of the CPN identity [12], later evidence indicates that SATB2 is required not only for CPNs, but also sub-cerebral projection neurons for their proper differentiation and axon pathfinding [80,133]. SATB2 and CTIP2 that respectively regulate the identity of callosal and sub-cerebral projection neurons are expressed in largely distinct neuronal populations during the prenatal period in layer V of the somatosensory cortex. However, the number of neurons co-expressing CTIP2 and SATB2 gradually increases after birth [47] (Figure 2). Neurons that postnatally co-express SATB2 and CTIP2 become two distinct neuronal subclasses projecting either contralaterally or to the brainstem, indicating that CTIP2/SATB2 co-expression plays a role in refining the neuronal property rather than specifying their identity [47]. Epigenetic modification of *Ctip2* locus by a transcriptional adaptor molecule LMO4 underlies the CTIP2 expression in SATB2-positive neurons in a time- and area-specific manner [47].

Transcription factor CUX1 controls the formation of callosal projections of layer II/III CPNs by regulating the expression of Kv1 voltage-dependent potassium channels, which in turn modifies activity-dependent axon development [134]. CPNs with reduced CUX1 expression still project to the contralateral hemisphere normally until P8, but result in impaired axonal innervation into the contralateral cortical plate due to the inability to turn on Kv1-dependent firing responses. Restoring CUX1 expression from P8 rescues both the axonal and electrophysiological defects in these neurons [134].

Together, these findings demonstrate that postnatal expression of specific transcription factors is essential not only for establishing unique anatomical and electrophysiological properties of CPNs, but also for the activity-dependent formation of callosal neural circuit.

#### 3.1.2. Other Players in CPN Specification

The roles of non-transcription-factor molecules in the development of callosal projections are also being characterized. The *Cav1* gene that encodes a membrane-bound scaffolding protein Caveolin 1 (CAV1) is one of the genes identified in the aforementioned microarray study that described many genes enriched in isolated embryonic and postnatal CPNs [63]. CAV1 is specifically expressed in a unique subpopulation of layer V CPNs that maintain dual ipsilateral frontal projections with expression peaking early postnatally [62]. Although CAV1 is not required for the specification of these CPNs or formation of dual axonal projections, its unique expression pattern suggests its role in maturation and refinement of these neurons [62].

Specific neurotrophic factors have been shown to control the survival of CPNs at distinct postnatal stages using in vitro cultures [135]. A recent in vivo study has further demonstrated that layer V CPNs express IGF1R, the receptor of insulin-like growth factor 1 (IGF1). The survival of layer V CPNs requires microglia-derived IGF1 acting as a trophic factor during early postnatal development [136]. The authors suggest that this is because these neurons do not receive sufficient trophic signals from their targets in the early postnatal period, as they have not yet penetrated or started branching in their targets. Although how these neurons acquire IGF1R expression is unknown, it highlights one aspect of their identity required during postnatal development.

### 3.2. Callosal Axon Guidance During the Postnatal Period

Callosal axons from lower cortical layers cross the midline during the embryonic period, while callosal axons from upper cortical layers reach the midline during the postnatal stage [92]. Although the mechanisms underlying the axon guidance through the midline during prenatal period have been extensively studied [9,86,96,100,102], the mechanisms of postnatal callosal axon guidance remains to be addressed.

Fate-restricted embryonic CPNs that are transplanted into the early-postnatal cerebral cortex can migrate into correct cortical layers and form appropriate callosal connections [131], suggesting that early postnatal cortex provides an environment similar to that in prenatal brain, for postnatal axon guidance. Alternatively, later growing callosal axons may utilize priorly crossed axons as their guides [56]. Currently, there is no clear evidence for guidance deficits that are stage-specific to postnatally growing axons, or postnatal-specific axon guidance mechanisms that dissociate from prenatal mechanisms.

By developing a new approach which allows identification and quantification of local transcriptomes and proteomes from labelled growth cones of single projections in vivo, Poulopoulos et al. obtained paired subcellular proteomes and transcriptomes from single CPN subtypes in the P3 mouse brain [137]. Such systems-level approaches may provide new findings of subtype- and stage-specific molecular mechanisms of callosal axon guidance.

After crossing the midline and reaching the target cortical area in the contralateral hemisphere, callosal axons make a turn from the white matter toward the cortical plate, arborize, and establish synaptic connections. These late processes largely depend on activity-dependent mechanisms, and have been extensively reviewed elsewhere [129,138].

## 4. Conclusions and Future Directions

The development of the CC is a long, well-orchestrated and dynamic process that requires precise control of many different signaling molecules and pathways. During the prenatal and postnatal periods, CPN specification and axon growth/guidance are essential. These relatively well-studied processes still have many mysteries to be solved. High throughput transcriptomics and proteomics of developing CPNs and CC tissue identified hundreds more possible molecular players that require further verification and functional characterization. Whole-brain spatial transcriptomics revealed new area- and layer-specific subregions within the adult mouse cerebral cortex [139]. Cross-referencing these new findings with previous gene expression data would further validate and expand current understanding of CPN identities. Most studies on the specification of CPNs have been focused on the events in postmitotic neurons. Since neuronal identities can also be controlled during progenitor cell stage, such mechanisms need to be further studied. For example, CPNs in lower layers and upper layers have been shown to differentiate from the same fate-restricted pool of neural progenitors expressing transcription factor CUX2, sharing a common lineage irrespective of their final layer position [140] (although the fate-restricted nature of CUX2^+^ progenitor cells remains under debate: [141,142,143]).

There is also a large gap in our understanding of some important processes of CC development at the molecular level. These include the mechanisms of topographic targeting of callosal axons, postnatal control of callosal axon guidance, and the mechanisms of the refinement of the CC that involves massive elimination of initially overproduced callosal axons and maintenance/preservation of specific axons. Postnatal CC development may have higher demands for intracellular protein transport, correct protein folding, post-transcriptional processing, and cellular energy regulation [126,144], but these hypotheses remain to be tested. A recent mouse study has demonstrated that the elimination of developmentally transient callosal projections during postnatal refinement is more massive than previously expected, and the pruning even includes transient callosal axons from layer IV neurons that never form callosal connections in the mature brain [92].

It is also important to keep in mind the possible differences between species, as recent advance in gene expression profiling reveals differences in gene expression in brain cells between humans and other species [145,146].

In summary, research on the development of callosal projection has entered a new phase at the molecular level, and is expected to develop and progress rapidly in the near future.

## Figures and Tables

**Table 1:** List of newly reported molecules that play roles in callosal projection neuron (CPN) specification.

Molecule	Molecular Function	Cortical Expression	CPN Subgroup Identification	Roles in CPN Development	References
CAV1	Lipid-bound scaffolding domain protein	Layer V in caudo-lateral cortex, late embryonic to early postnatal period	Dual projecting callosal/frontal projection neurons (CPN/FPN)	Not necessary for early specification of CPN/FPN; not necessary for dual axonal targeting; may function in postmitotic development and refinement	[62,63]
LMO4	Probable transcriptional factor	Layer V during early differentiation (E15.5), then expands to all cortical layer by P0 and later stages	CPNs and subcerebral projection neurons in presumptive sensory-motor area; colocalized with SATB2 in layer V by P6	Second backward projection development; molecular identity diversification of CPNs in rostral motor cortex	[73,74,75]
CITED2	Transcriptional coactivator of the p300/CBP-mediated transcription complex	Subventricular zone at E15.5; layer II/III, V, and VI in postnatal somatosensory cortex	CPNs in somatosensory cortex	Necessary for acquiring molecular identity of upper layer CPNs in somatosensory cortex	[11,63,76]
CTIP1	DNA-binding transcription factor	Embryonic callosal and corticothalamic projection neurons; high in all layers of somatosensory cortex and the most superficial aspect of layer II/III in motor cortex postnatally	Expressed by all CPNs	Repression of CTIP2 expression; specification of sensory area identity in CPNs and other neurons	[77,78,79]
FEZF2	DNA-binding transcription factor	Forebrain progenitors and their progeny in layer V	No	Repression of SATB2 expression; specification of subcerebral neuron identity	[48,60,80,81,82]
SNF2H	ATP-dependent chromatin remodeling protein	Embryonic neural progenitors	No	Primes upper layer cortical neuron development	[53,83,84]
INPP4B	Enzyme involved in phosphatidylinositol signaling pathways	TBD	No	Controlling axon polarization and generation of SATB2^+^ pyramidal neuron population	[54]
DOT1L	Histone methyltransferase specific to H3K79	Progenitor zone and cortical plate	TBD	Regulation of SATB2 and CTIP2 expression	[51,52,85]
ASCL1/NGN2	Basic helix-loop-helix family transcription factors	Neural progenitors in the embryonic ventral and dorsal telencephalon, respectively	No	Regulate the generation of SATB2^+^ upper layer neurons	[86,87,88]
FOXG1	Forked-head family transcription factor	Neural progenitors in embryonic cortex; high in layer II/III and lower layer V postnatally	No	Promotes SATB2 expression and layer II/III CPN specification; directly represses *Robo1* and *Slit3* expression; directly represses *Coup-TF1* expression	[66,68,89]
COUP-TF1	Member of nuclear hormone receptor family of steroid hormone receptors	Superficial cortical plate in embryonic brain; layer IV and upper layer V postnatally	No	Promotes layer IV identity, while suppresses layer II/III and layer V specification	[68]

**Table 2:** List of newly reported molecules that play roles in CPN axon guidance.

Molecule	Molecular Function	Cortical Expression	Interacting Pathway	Roles in Callosal Axon Development	Reference
APP	Receptor-like membrane protein	Embryonic and neonatal CC and neuronal cell body in layer V	Slit/Robo	Serves as a Slit receptor and mediates axon repulsion	[107]
HSC70	Molecular chaperone of the heat shock protein 70 (HSP70) family	Preferentially expressed in neurons	Netrin/DCC	Required for the stability of DCC/TRIO complex at the growth cone to mediate axon outgrowth and guidance	[108]
PLCγ1	Signal transducer of receptor tyrosine kinases	Broadly expressed in the brain from embryonic to adult stages; strongly expressed in the cortex	Netrin/DCC	Triggers actin rearrangement for axonal growth	[109]
MARCKS	Cellular substrate for protein kinase C; F-actin crosslinking protein	Ubiquitous	Netrin/DCC	Mediates DCC activation via membrane recruitment of tyrosine kinases PTK2 and SRC	[110,111]
GPM6A and GPM6B	Glycoprotein localized in cholesterol-rich lipid rafts of the plasma membrane	Expressed in actively elongating axons in embryonic and neonatal brain		Extension and guidance of callosal axons	[112,113]

## Data Availability

Not applicable.
